# Mass Spectrometry Imaging of Lipids in the Scent Glands of Muskrat (*Ondatra zibethicus*) in Different Reproductive Statuses

**DOI:** 10.3390/cells11142228

**Published:** 2022-07-18

**Authors:** Wenqian Xie, Shengheng Mu, Jinkun Zhong, Chaoran Zhang, Haolin Zhang, Xiaodong Wang, Qiang Weng

**Affiliations:** 1 Laboratory of Animal Physiology, College of Biological Sciences and Technology, Beijing Forestry University, Beijing 100083, China; xwq11@bjfu.edu.cn (W.X.); mushengheng@bjfu.edu.cn (S.M.); zhongjinkun2002@bjfu.edu.cn (J.Z.); zhangcr@bjfu.edu.cn (C.Z.); 2 Key Laboratory of Mass Spectrometry Imaging and Metabolomics, Minzu University of China, State Ethnic Affairs Commission, Beijing 100081, China; xiaodong@muc.edu.cn; 3 Centre for Imaging & Systems Biology, College of Life and Environmental Sciences, Minzu University of China, Beijing 100081, China

**Keywords:** lipid metabolism, MALDI-MSI, seasonal changes, scent gland, muskrat

## Abstract

As a typical seasonal breeding animal, male muskrats have a pair of scent glands that can emit musky odor substances to attract females during the breeding period. The present study aimed to visualize the differences in the distribution of lipids in the scent glands of muskrats during their different reproductive statuses by imaging mass spectrometry and quantitative real-time PCR (qRT-PCR). The results revealed remarkable differences in the expression and spatial distribution of lipids detected in the scent glands of muskrats during the different reproductive statuses. In addition, the expression levels of lipid molecules PC (32:0) and LysoPC (16:0) were found to be significantly higher in the breeding season than in the non-breeding season. Moreover, the mRNA expression levels of lipid synthesis enzyme *Pemt* and *Pla2g4b* were higher in the breeding season than in the non-breeding season, and there were positive correlations between the expression intensities of lipid molecules and the expression levels of *Pemt* and *Pla2g4b*. The present study investigates the changes and distribution of the endogenous lipid in the scent glands of muskrats and elucidates that the seasonal changes in the lipid metabolism may affect the functions of the scent glands in muskrats.

## 1. Introduction

Lipids are a class of compounds that are readily soluble in organic solvents and are non-homogeneous in chemical composition and structure. Lipids are essential organic compounds that fulfill a fundamental role as the structural components of cell membranes. Additionally, lipids play a role as energy stores, sources of signaling molecules and signal transducers [1,2,3]. Several studies have reported that the main lipids present in the biological membranes of mammalian cells consist of several lipid categories, including triacylglycerols, sphingolipids, fatty acyls, glycerolipids and sterols [4,5]. The structures of lipids are complex and varied among species, suggesting that different lipids play enormous and vital roles in various critical biological functions [6,7,8]. Recent publications have reported that lipids regulate various cellular and physiological processes, including energy conversion, transport, information recognition and transmission, cell development, differentiation and apoptosis [3,7]. Lipids are also involved in hormonal signaling pathways that exert essential effects on the seasonal fluctuations in vertebrates [9,10]. The study of lipids has developed into a research field of increasing importance, which is expanding rapidly due to the identification of new lipids and their multiple biological functions.

Lipidomics is a novel technology that can be used to analyze and determine the lipids and metabolites of organisms, cells, and tissues, to elucidate the structures and functions of lipid metabolism [11,12]. Over the last decade, mass spectrometry imaging (MSI) has become a promising technique which is able to investigate the spatial distribution of a variety of molecules in different tissues and cells [13,14,15]. In recent years, matrix-assisted laser desorption/ionization-mass spectrometry (MALDI-MS), secondary ion mass spectrometry (SIMS) and desorption electrospray ionization (DESI) have been used as the main and well-established MSI techniques in biological fields [16]. MALDI-MS is the common microprobe technique used to straightforwardly detect and map numerous biomolecules from the tissue sections [17]. In brief, a thin layer of a specific matrix was applied to the surface of slices or sections of tissue, followed by sequential mass spectrometry (MS) on the sample. Based on the MS information in each pixel of the samples, the distribution images of hundreds of molecules would be generated simultaneously [18]. MALDI-MS imaging has been widely applied to the imaging of endogenous molecules, including amino acids, nucleotides, fatty acids, and lipids in biological and clinical samples [18,19]. There have been increasing applications of MALDI-MS to map the distribution of metabolites and proteins in mammal tissues with high sensitivity and specificity [15,20,21,22]. This method is highly streamlined and offers a general understanding of the in situ spatial distribution of lipids by MALDI-MS in mammals.

The muskrat (*Ondatra zibethicus*) is a kind of herbivorous rodent that lives in water that are local to North America [23]. Considered a typical seasonal breeding animal, the adult male muskrat undergoes a sexually active period from March to October, known as the breeding period [24]. The male muskrat can discharge musky odor substances via its scent glands, situated in the tail between the skin and muscle during the breeding period [25,26]. However, during the non-breeding period, the scent glands shrink and secrete slight musky odor substances or no musky odor substances [27]. Numerous studies have reported that hormones regulate the reproductive state of rodents and hormonal control can manage the rodent population at the landscape scale [28]. Previously, the levels of steroid hormones in the serum and the scent glands have been consistent with the seasonal morphological variation of the scent glands, thus suggesting that steroid hormones may regulate the seasonal changes in the scent glands [29,30,31]. Several studies have reported that the main components secreted by the scent glands are esters, fatty acids, cyclic ketones and steroids, which are similar to the components of Moschus [32,33,34]. Numerous macrocyclic ketones and long-train fatty acids have been detected in the scent gland of the muskrat using gas chromatography/mass spectrometry (GC-MS) [35]. Transcriptome sequencing results have suggested that the differing expressed transcripts of the scent glands in muskrats are mainly enriched in fatty acid metabolism, fatty acid biosynthesis and fatty acid degradation, suggesting that the seasonal changes in scent glands are related to lipid synthesis [36,37]. The differential metabolites and metabolic pathways of the scent glands during the different reproductive statuses remain unclear.

The aim of this study is to examine the distribution of various metabolites in situ and the synthesis of metabolites including fatty acid in the scent glands and lipid metabolic pathways that regulate the functions of scent glands during the different reproductive statuses. In addition, the study is the first to explore the spatial distributions of metabolites in the scent glands of this seasonal breeding animal and the role of lipid metabolic pathways in the regulation of scent gland function. The results will assist our comprehension of the endogenous metabolism compounds, along with the regulation of the lipid metabolic pathway in the scent glands of muskrats during the different reproductive statuses.

## 2. Materials and Methods

### 2.1. Animals

All animal experiment procedures followed the procedures recommended by the Experimental Animal Ethics Committee of Beijing Forestry University. All animals were obtained in May (the breeding season, n = 10) and December (the non-breeding season, n = 10) from Xinji Muskrats Breeding Farm, Hebei Province, China. After the muskrats were paralyzed using diethyl ether, the scent glands were quickly dissected from the male muskrats and immediately frozen at −80 °C prior to the MS experiment and the molecular experiments.

### 2.2. Tissue Collection

Twenty-micrometer thick sections were cut from the frozen scent glands of muskrats with an optimal cutting temperature compound (OCT, Sakura Finetek USA, Inc., Torrance, CA, USA) using a Leica CM1100 cryostat (Leica Microsystems, Inc., Wetzlar, Germany) and collected onto the indium–tin oxide-coated microscope glass slides (ITO slides, 80 Ω, 2.5 × 7.5 cm, Bruker Daltonics, Berman, Germany). Before the matrix coating, the tissue sections were dried at room temperature and captured on an Epson Perfection V550 Photo Scanner (Seiko Epson Co., Tokyo, Japan) to obtain histological images for the correction and calibration of subsequent mass spectrometry image acquisition area.

### 2.3. MALDI-TOF/TOF MS/MS Analysis

The 2-Mercaptobenzothiazole (2-MBT) was dissolved at a concentration of 12 mg/mL in a blended acetonitrile: water: TFA (80:18:2, *v*/*v*/*v*) solution. Serial tissue sections of the scent glands of muskrats were executed with the 2-MBT matrix using a Bruker Daltonics ImagePrep matrix electronic sprayer (Bremen, Germany). The matrix coatings for each grid were made of a 2 s splash, a 30 s brooding, and a 60 s drying for each spray cycle, 40 splash cycles were performed [15]. The sections were sprayed with a second cycle of the matrix solution until good MALDI-MS were obtained across the whole sections. All MS data were reserved on the Bruker MALDI-TOF-MS (Bruker Daltonics, Berman, Germany) equipped with a solid-state Smartbeam Nd: YAG UV laser (355 nm, Azura Laser AG) which was employed in the positive-ion linear mode [15]. All the mass spectra were acquired over a mass range of *m/z* 150 to 3500 in positive-ion mode with broadband detection. For the acquisition of MALDI-MS profiling data, the mass spectra were recorded from an accumulation of 20 laser scans, and each scan was accumulated from 500 laser shots. The *m/z* values of the ions of the following compounds were used for external mass calibration: PC (32:0) ([M + Na]^+^, *m/z* 756.6), Lyso (16:0) ([M + H]^+^, *m/z* 496.3). Laser impulse energy was approximately 180 mJ, and the laser repetition rate was 2000 Hz [15]. For MS profiling data analysis, the Bruker FlexAnlysis 3.4 software was performed for mass spectral viewing and processing. The Bruker FlexImaging 4.1 software was applied to reconstruct the mass spectral data. In brief, a mass window of 0.3% and a signal to noise (S/N) ratio of 3 were selected for peak lists’ generation to the sum of all pixels. The values of height, peak width and minimum intensity threshold were set as default. All spectra were manually confirmed. For the database searching, three ion adduct forms (i.e., [M + H]^+^, [M + Na]^+^, and [M + K]^+^) were considered. With the same conditions, signal lists of all pixels in the scent glands during the breeding and non-breeding season were obtained in three separate repetitions of the experiment.

### 2.4. Metabolites Extraction

A total of 50 mg of scent gland tissue was weighed and added to 1000 μL extraction solvent containing internal standard and concertation of 2 μg/mL (methanol: acetonitrile: water= 2:2:1) and then placed in an Eppendorf (EP) tube. EP tube was put in ice water and a ball mill was added for 4 min at 45 Hz, then ultrasound for 5 min. After grinding 3 times, the sample was placed for 1 h at −20 °C and centrifuged at 12,000 rpm for 15 min at 4 °C for protein precipitation. A total of 825 μL of supernatant was placed in a new EP tube and the extract was dried in a vacuum concentrator. A total of 100 μL mixed extraction solvent (V acetonitrile: V water= 1:1) was added to the dried metabolite, vortexed for 30 s, and then put into ice water for ultrasonic treatment for 10 min. After ultrasonic treatment, the tissues were centrifuged at 12,000 rpm, 4 °C for 15 min. A total of 75 μL of supernatant was transferred into 2 mL LC/MS glass vial. Quality control (QC) samples were mixed among the six samples and 75 μL was taken for the UHPLC-QTOF-MS/MS analysis reconstitution.

### 2.5. LC-MS/MS Analysis

The UHPLC system (1290, Agilent Technologies, Santa Clara, CA, USA) was utilized to perform the LC-MS/MS analyses. A 1.7 μm 2.1 × 100 mm of UPLC BEH Amide column (Waters Corporation, Milford, MA, USA) was placed into TripleTOF 6600 (AB Sciex, Framingham, MA, USA). The data acquisition for MS and MS/MS analysis was carried out using the Analyst TF 1.7 software (AB Sciex, Framingham, MA, USA). In each cycle, 12 precursor ions above *m/z* 100 were chosen for the acquisition of fragmentation to collision energy (CE) of 30 eV. ESI source conditions were set as follows: ion source gas 1 as 60 Psi, ion source gas 2 as 60 Psi, curtain gas as 35 Psi, source temperature 600 °C, ion spray voltage floating (ISVF) 5000 V in positive ion mode. The MS data were transformed by ProteoWizard software, and XCMS was used for proofreading. The R package CAMERA and mass spectrometry database were used for metabolite identification and enrichment pathways.

### 2.6. GC-MS Analysis

The odor substance samples from scent glands were added to neat ethanol. The muscone (B21154, Shanghai Yuanye Bio-Technology Co., Ltd., Shanghai, China) was set as standard sample for observation. The separation and detection of analytes were achieved with a TurboMatrix HS 40 Clarus SQ 8 GC/MS (PerkinElmer, Inc., Waltham, MA, USA). The chromatographic column was an HP-5MS capillary column (30 m × 0.25 mm, 0.25 μm). The separation procedure of GC system was set at the initial oven temperature 100 °C holding for 7.5 min, then heated up to 250 °C at the speed of 35 °C/min, and held for 0.7 min, then ramped up to 300 °C at the speed of 40 °C/min, finally holding for 0.5 min. The temperature of injection port, ion source, and transfer line were all set at 250 °C. Helium was the carrier gas with the flow rate 1.5 mL/min. The split mode was set with the ratio of 1:5. The peak comparison was performed by Xcalibur software, matching with the NIST 05a.L spectral library database.

### 2.7. H&E Staining

After MALDI-TOF/TOF MS/MS experiments, the scent gland sections of the breeding and non-breeding periods were washed with ethanol series to remove the matrix. Some sections, which were embedded in paraffin and OCT compound, were dyed with hematoxylin-eosin (H&E) for histological observation.

### 2.8. Quantitative Real-Time PCR (qRT-PCR)

Following the manufacturer’s protocol, the extraction of RNA from each scent gland was utilized TRIzol Reagent (Invitrogen Co., Carlsbad, CA, USA) [37]. Firstly, StarScript II First-strand cDNA Synthesis Mix (GenStar, Beijing, China) was used to synthesize the extracted RNA into cDNA for reverse transcription. In brief, 10 μL of the reaction mixture comprised 3 μL of cDNA, 5 μL of Universal Blue qPCR SYBR Green Master Mix (11184ES03, Yeasen Biotechnology Co., Ltd., Shanghai, China), 0.1 μL of primer sequences, 1.8 μL of ddH_2_O. Primer sequences for qRT-PCR are listed in Table 1. The qRT-PCR procedure was executed in ABI PRISM 7500 Fast Real-Time System (Applied Biosystems, Foster City, CA, USA), including 95 °C preheating for 10 min and followed by a denaturation period of 30 s at 95 °C, annealing period of 30 s at 60 °C and extension period of 30 s at 72 °C. After 40 cycles, the melting curves were employed to check the homogeneity of products. Negative control was set for each experiment and samples were run 3 times to ensure the intra-assay variation was less than 10%. The relative value was calculated by the 2^−ΔΔCq^ method using the *Actb* mRNA expression. All experiments were performed in triplicate and repeated 3 times.

### 2.9. Statistical Analysis

Statistical analyses were employed and analyzed using the Student’s *t*-test and GraphPad Prism 7.0 (GraphPad Software Inc., San Diego, CA, USA). *p* < 0.05 was regarded as a significant difference.

## 3. Results

### 3.1. Anatomic Localization and Tissue Sections of the Scent Glands of Muskrats

**Figure 1:**
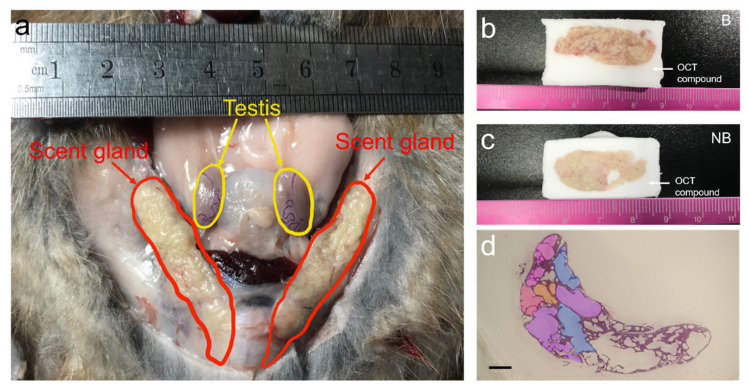
Anatomic localization and whole-tissue sections of the scent glands in muskrats during the breeding and non-breeding seasons. (**a**) The anatomic localization and morphology of the scent glands; (**b**,**c**) the scent glands held into position using OCT compound during breeding and non-breeding seasons; (**d**) the cross-section of scent glands. The purple section represents large duct, blue section represents medium duct, orange section represents small duct, pink section represents fine duct. B, the breeding season; NB, the non-breeding season. Scale bars = 125 μm.

Tissue sections and anatomic localization of the scent glands in the muskrats are shown in Figure 1. As shown in Figure 1a, the scent glands are situated between the feather layer and muscle layer at the ventral base of the tail. The whole sections of the scent glands using an OCT compound are shown in Figure 1b,c. A cross-section of the scent glands is shown in Figure 1d. According to the size of the pipe diameter, the fragrant ducts of the scent glands were divided into the fine duct, the small duct, the medium duct, and the large duct. All levels of the duct were connected successively.

### 3.2. Main Compounds Based on LC-MS/MS and GC-MS

**Figure 2:**
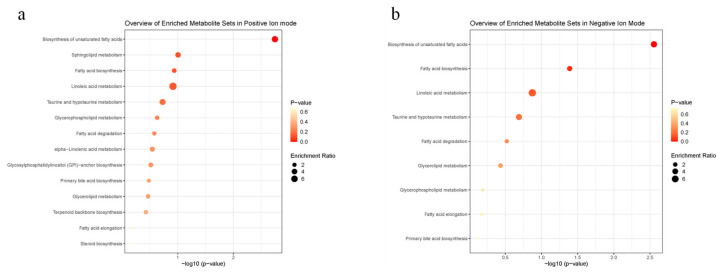
KEGG enrichment analysis of lipids metabolites sets detected from scent glands by LC-MS/MS. (**a**) Overview of enriched metabolite sets in positive ion mode; (**b**) overview of enriched metabolite sets in negative ion mode.

As shown in Figure S1 and Table S1, the main compounds of scent glands by GC-MS include cyclotridecanone, Z-7-hexadecenoic acid, cyclopentadecanone, cyclohexanecarboxylic acid and so on. Combining the GC-MS data and previous reports, the main compounds secreted by scent glands might be macrocyclic ketones and long-train fatty acids. Using *p* < 0.05 and VIP (variable importance in the projection) > 1 as selection thresholds, the 98 and 49 differentially expressed metabolites between the breeding seasons and the non-breeding seasons in both positive and negative ion modes were shown in Table S2 and Table S3, respectively. Those metabolites could be clarified as lipids and lipid-like molecules, carboxylic acids and derivatives and so on. The ten main differentially expressed lipids compounds detected by LC-MS/MS are shown in Table 2 and Table 3. As shown in Figure 2, Kyoto Encyclopedia of Genes and Genomes (KEGG) enrichment analysis in both positive and negative ion modes revealed that differentially expressed lipids in scent glands were mainly enriched in the biosynthesis of unsaturated fatty acids, fatty acid biosynthesis and glycerophospholipid metabolism. The results of differentially expressed metabolites by LC-MS/MS found a possible association of fatty acids with seasonal changes.

### 3.3. Principal Component Analysis (PCA) Based on MALDI-TOF/TOF MS/MS

**Figure 3:**
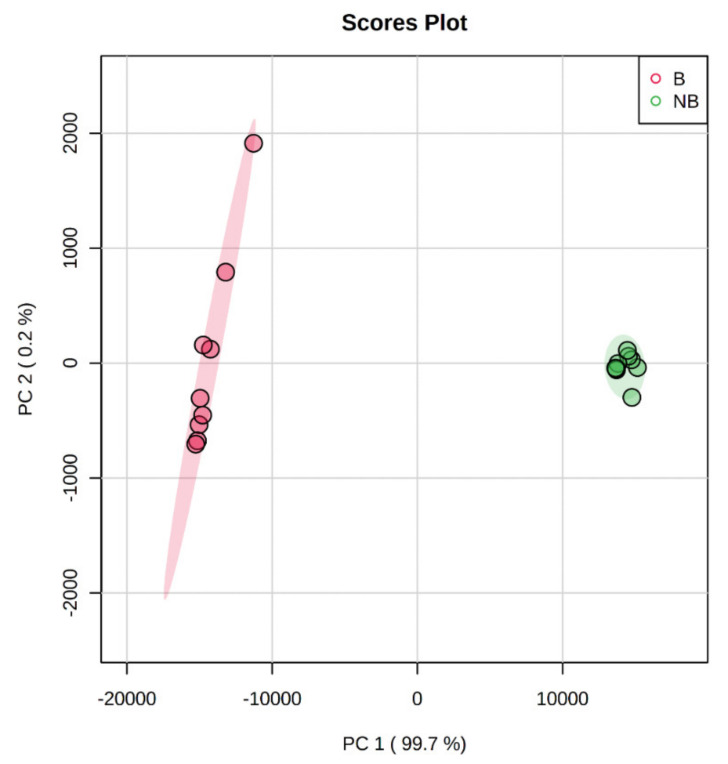
PCA score plot of metabolites detected from scent glands during different reproductive statuses. B, the breeding season; NB, the non−breeding season.

A PCA score plot was constructed based on the MALDI-MS profiling dataset to investigate differences in the scent glands between the breeding and the non-breeding seasons. As presented in Figure 3, a clear separation between the breeding and non-breeding seasons on the axis of the first principal component (PC1) (99.7%) and the second principal component (PC2) (0.2%) was observed, which explained 99.8% of the total variance. The metabolites in the scent glands of the breeding season were clearly separated from those in the non-breeding season. In addition, the metabolomic alterations over the breeding season were greater than those in the non-breeding season.

### 3.4. MALDI-MS In Situ Detection and Imaging of Different Metabolites in the Scent Glands of Muskrats during Different Reproductive Statuses

**Figure 4:**
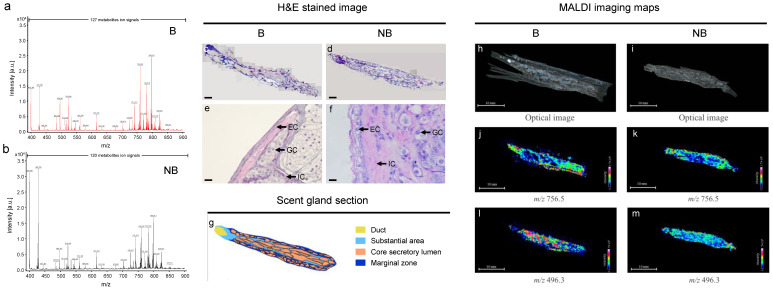
In situ detection and imaging of different metabolites from scent glands of muskrats during the breeding season and the non-breeding season by MALDI-TOF MS in the positive ion mode. (**a**,**b**) The total ion current obtained for the whole tissue sections by MALDI-TOF MS in the positive ion mode during the breeding and non-breeding seasons. (**c**–**f**) Histological observation of the scent glands during the breeding and non-breeding seasons. (**g**) Diagram of the scent glands based on histological structure, including duct, substantial area, core secretory lumen and marginal zone. (**h**,**i**) The 20 μm tissue sections of the scent glands by MALDI-TOF MS in the positive ion mode during the breeding and non-breeding seasons. (**j**,**k**,**l**,**m**) MALDI-TOF MS imaging maps of the ion at *m/z* 756.5 and *m/z* 496.3 detected in the scent glands during breeding and non-breeding seasons. B, the breeding season; NB, the non-breeding season.

The ion signals of the metabolite substances, as well as the distribution of selected metabolites in the scent glands during the breeding and non-breeding seasons, are shown in Figure 4. The ion signals in the scent glands of muskrats using MALDI-MS are represented in Figure 4a,b. A total of 127 and 120 ion signals were detected in the breeding season and the non-breeding season, respectively. Among them, a total of 106 ion signals were observed in both seasons, while 21 and 14 ion signals were detected in the breeding and non-breeding seasons, respectively. Histological observations of the scent glands in muskrats during the breeding and non-breeding periods are exhibited in Figure 4c–f. Based on the histological observations, a diagram of the scent gland is presented in Figure 4g. As shown in Figure 4c,d,g, the whole scent gland tissue sections of a male muskrat were stained by H&E staining to show distinguishable compartments of the scent gland structure, including the duct, the core secretory lumens, and a substantial area consisting of various types of cells and the marginal zone. Histological observations in the substantial area of the scent glands of muskrats during the breeding and non-breeding seasons are shown in Figure 4e,f. Epithelial cells, glandular cells and interstitial cells were observed in the scent glands during different reproductive periods.

To detect differential metabolite identification, two tandem MS analytical approaches were employed in this work. In brief, the distribution of metabolites on tissues was revealed using MALDI-TOF/TOF MS/MS analysis, while metabolites in tissues were further confirmed using LC-MS/MS analysis as a complementary identification strategy. The MS/MS analysis at *m/z* 496.3 and *m/z* 756.5 from MALDI and LC are shown in Figures S2–S5. As shown in Table 4 and Figures S2–S5, the structurally specific fragment ions of the differential metabolites detected by the MALDI-TOF/TOF MS/MS and LC-MS/MS were highly consistent, strengthening the reliability of the identification of these metabolites. Within the experimental error, the ion signal of *m/z* 756.5 and *m/z* 496.3 detected by MALDI-MS/MS was identified as PC (32:0) and LysoPC (16:0) by matching with LC-MS/MS database, respectively. The sagittal sections of 20 μm thickness were cut in a biological specimen cryostat (Figure 4h,i). To show the metabolites for MALDI imaging, the distribution of selected metabolite ion signals with lipids (i.e., *m/z* 756.5 and *m/z* 496.3) in scent gland tissue sections were determined in Figure 4j,k,l,m. The MALDI-MS imaging maps of the ion at *m/z* 756.5 were mainly located in the ducts and marginal zone of the scent glands during different reproductive statuses. Moreover, MALDI-MS imaging maps of the ion at *m/z* 496.3 were mainly located in the core secretory lumens of the scent glands in both seasons.

### 3.5. Schematic Diagram of Metabolic Pathway and Expression Diagram of Metabolite Synthase

**Figure 5:**
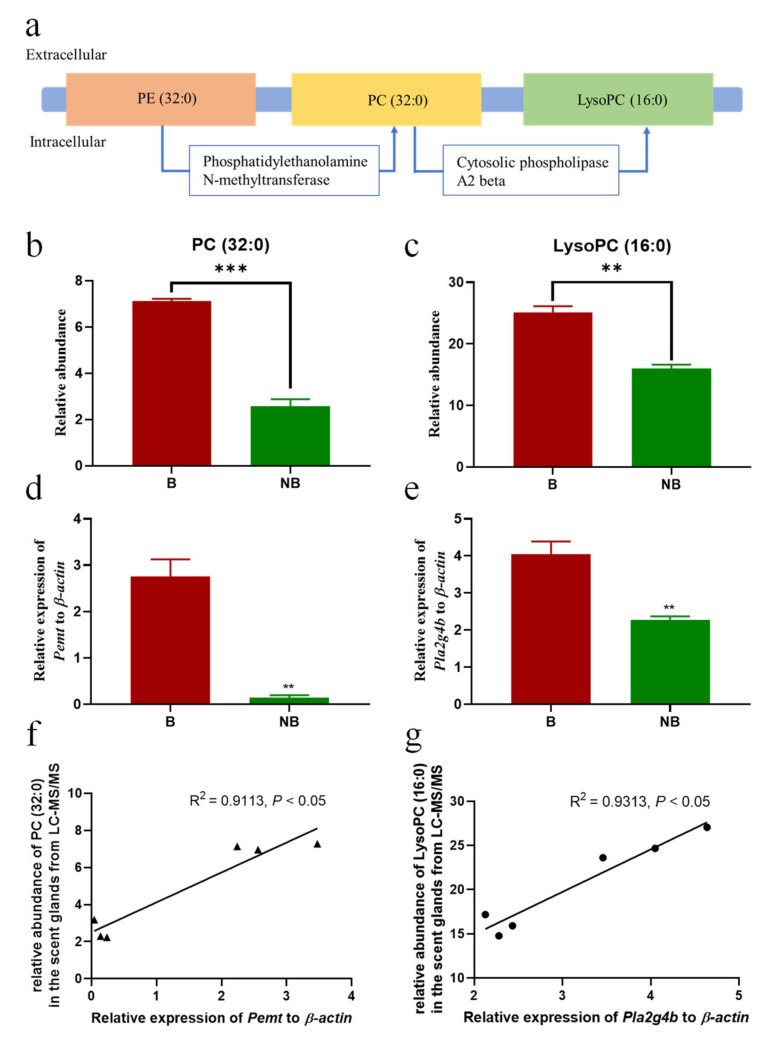
Schematic diagram of metabolic pathway and expression histogram of metabolite synthase. (**a**) A schematic diagram showing membrane-localized PC (32:0) and LysoPC (16:0) and the enzyme mediating the conversion of compounds in the scent glands of muskrats. (**b**,**c**) Relative quantification results for PC (32:0) and LysoPC (16:0) in the scent glands detected by LC-MS/MS during the breeding and non-breeding seasons. (**d**,**e**) Relative expression of *Pemt* and *Pla2g4b* in the scent glands of muskrats during the breeding and non-breeding seasons. (**f**,**g**) The linear correlation and scatter diagrams of relative expression of *Pemt* and *Pla2g4b* and relative abundance of PC (32:0) and LysoPC (16:0) of the scent glands, respectively. Each dot means the relative mRNA levels and relative abundance of PC (32:0) and LysoPC (16:0) during the breeding season (n = 3) and the non-breeding season (n = 3). B, the breeding season; NB, the non-breeding season; Data represent the mean ± SEM.; * statistically significant values (** *p* < 0.01; *** *p* < 0.001).

The metabolic pathway schematic diagram and expression histogram of the PC (32:0) and LysoPC (16:0) in the scent glands of muskrats (Figure 5). As shown in Figure 5a, PC (32:0) was mainly synthesized by phosphatidylethanolamine-N-methyltransferase (PEMT) using PE (32:0) as a substrate in the cell membrane, and LysoPC (16:0) was formed by the hydrolysis of PC (32:0) catalyzed by cytosolic phospholipase A2 beta (PLA2G4B). The relative abundance of PC (32:0) in the scent glands detected by LC-MS/MS is shown in Figure 5b. The relative abundance of PC (32:0) in the scent glands was significantly higher in the breeding period than in the non-breeding period. The expression of the critical enzymes in the breeding period was higher than that of the non-breeding period (Figure 5d). The linear correlation and scatter diagram of relative expressions of *Pemt* and the relative abundance of PC (32:0) from LC-MS/MS during the breeding and non-breeding seasons are shown in Figure 5f. A scatter diagram of the relative expression of *Pemt* shows positive correlations with the relative abundance of PC (32:0) (r = 0.9113, *p* < 0.05). Meanwhile, the relative abundance of LysoPC (16:0) detected in LC-MS/MS in the scent glands is shown in Figure 5c. The abundance of LysoPC (16:0) in the scent glands was significantly higher in the breeding season than in the non-breeding season. The expression of critical enzymes *Pla2g4b* in the breeding period was higher than that of the non-breeding period (Figure 5e). A scatter diagram of relative expression of *Pla2g4b* shows positive correlations with the relative abundance of LysoPC (16:0) (r = 0.9313, *p* < 0.05).

## 4. Discussion

In the study, we found that the differentially expressed metabolites in the scent glands of muskrats were mainly enriched in lipid metabolism. Furthermore, we demonstrated the in situ distribution of endogenous lipid molecules in the scent glands of muskrats. The results showed that the expression levels of lipid molecules PC (32:0) and LysoPC (16:0) were significantly different in the scent glands under the compound correlated analysis during the breeding and non-breeding seasons. It was also consistently found that the mRNA expression levels of the *Pemt* and *Pla2g4b* were significantly different in the scent glands. The present study suggested that lipid synthesis might be involved in regulating the seasonal changes in the morphology and functions of the scent glands in muskrats during the breeding and non-breeding periods.

In vertebrates, lipids are widely found and act as key signal transducers and hormones regulating a variety of cellular and physiological processes, from metabolism and cell death to inflammation and immune responses [38]. LC-MS/MS analysis and MALDI-MSI are the crucial methods by which to identify the differentially abundant lipid metabolites and reveal the spatial distribution in various types of biological tissues [39,40]. In the present study, 98 and 49 differentially expressed metabolites were identified in the scent glands in the breeding and non-breeding seasons by LC-MS/MS analysis in the positive and negative ion modes, respectively. Consistently, 127 and 120 ion signals were also observed by MALDI-TOF MS in the scent gland during the breeding and non-breeding periods. In addition, a clear separation was found between the breeding and non-breeding seasons on PC1 and PC2 axis with the total variance based on MALDI-TOF MS results. Combining those data, it showed that the expressions of endogenous metabolites in the scent gland were significantly different and closely related to seasonal changes. As indicated by GC-MS and a previous report, lipid molecules including macrocyclic ketones, fatty acids and esters are the main substances secreted by the scent gland [32]. Furthermore, the KEGG pathways are enriched in the biosynthesis of unsaturated fatty acids, fatty acid biosynthesis and glycerophospholipid metabolism. These data indicate that lipid metabolites might exert their significant roles in the scent glands of muskrats. In addition, 9-octadecenoic acid was observed to be significantly decreased by silencing lipid-related enzymes ACOX3 and HSD17B4 genes in the scent glands of muskrats, thus indicating that the content of lipid substances in the scent glands of muskrats could be regulated by the peroxide metabolism [41]. Meanwhile, our previous reports showed that the seasonal expression differences in steroid synthases SF-1, StAR, P450scc, P450c17 and steroid hormone receptors in the scent glands of muskrats indicated that the scent glands had steroid synthesis abilities, and that the steroid hormones regulated the functions of the scent glands in the breeding and non-breeding periods [25,42]. The significant correlation between the levels of androgens and estrogens and musk composition in musk deer (*Moschus berezovskii*) suggested that sex steroid hormones regulate musk secretion by influencing lipid synthesis [43]. Taken together, the results suggest that endogenous lipid metabolites might be involved in regulating the seasonal changes in scent gland functions during the different reproductive statuses.

Phosphatidylcholine is widespread in animals and is one of the main components of phospholipid molecules [44,45]. In the present study, the differentially expressed metabolites enriched in one of the lipid metabolism pathways, glycerophospholipid metabolism, based on LC-MS/MS analysis. Meanwhile, the results showed that the ion signals of PC (32:0) and LysoPC (16:0) molecular species differed in the scent glands during the breeding and non-breeding periods, suggesting that the PC (32:0) and LysoPC (16:0) were the main compounds of the musky odor substances in the scent glands of muskrats. The distribution of PC (32:0) and LysoPC (16:0) was observed in the secretion area of the scent glands in the breeding period, thus indicating that phospholipids might be involved in regulating the functions of scent glands during the breeding and non-breeding periods. Several publications reported that the accumulation of phosphatidylcholine could promote nerve conduction, increase membrane enzyme activity, and regulate the growth of animals [45,46]. A variety of PC (32:0) has been identified as a diagnosis marker. PC (16:0/16:0) was one of the important pulmonary surfactants and was observed to be increasingly high in the damaged cranial nerve, thus participating in the cytomembrane of cells [47]. PC (16:0/16:0) was identified as a marker for pneumonia or lung cancer diagnosis due to the immune response with the anti-SLC34A2 antibody, which was a specific marker of type II alveolar epithelial cells [46]. Furthermore, large amounts of free fatty acids and steroids were detected in the preputial glands of musk deer [48]. In combination with the results of the present study, the accumulation of PC (32:0) during the breeding season suggested that PC (32:0) was a potential marker of the muskrats’ musk.

Glycerol phospholipid metabolic pathways are widespread in vertebrates, particularly in the composition and function of membrane lipids [7,49,50]. Phosphatidylcholine synthesis was performed from the available choline using the cytidine diphosphocholine (CDP-choline) pathway or PE methylation pathway [51,52]. In brief, the synthesis of PC (32:0) could occur via the substrate PE (32:0) and catalyzed using PEMT [53]. LysoPC (16:0) was previously synthesized through the hydrolysis of PC (32:0) with the PLA2G4B enzyme [54]. LysoPC (16:0) acts as a biological characteristic of extracellular growth factors or signaling molecules, and participates in the physiological processes of organisms by activating the downstream signaling pathway [55]. In the present study, the expression pattern of *Pemt* and the distribution of endogenous molecule PC (32:0) in the breeding period were higher than those in the non-breeding period. Meanwhile, positive correlations between the expression level of *Pemt* and the relative abundance of molecular PC (32:0) were consistent, indicating that the synthesis of PC (32:0) was related to seasonal changes, and that PC (32:0) might be involved in regulating the functions of the scent glands. The expression patterns of *Pla2g4b* and LysoPC (16:0) were the same as those of *Pemt* and PC (32:0). Previous studies had reported that the glycerophospholipid metabolic pathway was closely related to the energy metabolism of animals [49,56]. A significantly higher proportion of unsaturated fatty acids was observed in the brown bear (*Ursus arctos*) during winter, implying that the metabolic pathway of lipids may be involved in seasonally specific metabolic regulation [57]. Consistently, the differences in phospholipid fatty acid profiles have consistently been observed to be involved in the regulation of the functions and energy metabolism in Syrian hamsters (*Mesocricetus auratus*) and the garden dormouse (*Eliomys quercinus*), indicating that phosphorylation metabolic pathways might be involved in the maintenance of lipids during torpor [58,59,60]. Altogether, the results of this study showed that the scent glands might be a target organ of the glycerophospholipid signaling pathway, and that the glycerophospholipid metabolic pathway might regulate the functions of the scent glands in the breeding and the non-breeding periods.

## 5. Conclusions

The present study revealed that there were significant differences in the metabolites of the scent glands during the breeding and non-breeding seasons. The PCA results revealed that the difference in metabolites might be related to seasonal morphological changes. The endogenous molecules and the in situ positioning situation of PC (32:0) and LysoPC (16:0) suggested that endogenous metabolites regulated the morphological changes in the scent glands. The mRNA expression of *Pla2g4b* and *Pemt* further suggested that the phospholipid metabolic pathways regulated the function of the scent glands. This is the first time that the distribution of endogenous lipid molecules has been shown in situ, as well as the synthesis of phospholipid metabolic pathways in the scent glands of muskrats.

The present data effectively provide a greater understanding of the spatial distribution and metabolism of specific lipids in the scent glands of muskrats during the breeding and non-breeding periods. The abundance, spatial distribution as well as metabolic enzymes of PCs and LysoPCs are highlighted in the present study. However, we also observed the significantly different abundance of other lipids or lipid-like molecules, carboxylic acids and derivatives in the scent glands during the different statuses. The anabolic and catabolic pathways and potential functions of those metabolites in the scent glands of muskrats remain unclear. In the future, we will further explore the metabolic pathways and functions of musk substances in muskrats’ musk, aiming to provide new insights into the understanding of seasonal variation in the metabolic landscape of scent glands in muskrats.

## Figures and Tables

**Table 1:** The primer used for quantitative real-time PCR amplification.

Gene Name	Primer Sequence (5′->3′)	Product Length (bp)
*Pemt*	F: CCACATTTCCCTTCAGCGTG	175
	R: CGGTAGATCTCCGCAGTGAA	
*Pla2g4b*	F: GTGTTGAGCATGACTAGATCCCA	191
	R: AGACCACTCTGGGTCCTCAT	
*Actb*	F: GACTCGTCGTACTCCTGCTT	223
	R: AAGACCTCTATGCCAACACC	

**Table 2:** Identification of differentially expressed lipids detected in the scent glands during the breeding and non-breeding seasons in the positive ion mode of LC-MS/MS.

Compound Name	*m/z*	RT (Min)	Mean B	Mean NB	*p* Value	Fold Change
PC(16:0/16:0)	756.525	2.354	7.124	2.568	0.031	1.791
Phosphatidylethanolamine	748.571	2.122	0.167	0.097	0.025	1.723
1-Stearoyl-2-arachidonoyl-sn-glycerol	627.529	3.108	0.085	0.134	0.000	0.634
LysoPC(16:0)	496.313	3.026	25.106	15.954	0.028	2.493
Glycochenodeoxycholate	467.351	3.098	0.029	0.009	0.048	3.279
Nervonic acid	430.375	2.748	0.171	0.100	0.015	1.700
Stearoylcarnitine	428.370	2.543	12.765	7.198	0.001	1.773
16-Hydroxypalmitic acid	314.266	0.743	0.095	0.055	0.007	1.727
Linoleic acid	298.271	0.603	0.552	0.303	0.046	1.821
sn-Glycerol 3-phosphoethanolamine	216.061	6.404	0.238	0.080	0.032	2.967

**Table 3:** Identification of differentially expressed lipids detected in the scent glands during the breeding and non-breeding seasons in the negative ion mode of LC-MS/MS.

Compound Name	*m/z*	RT (Min)	Mean B	Mean NB	*p* Value	Fold Change
L-palmitoylcarnitine	458.348	2.652	0.610	0.225	0.002	2.718
1-Oleoyl-sn-glycerol 3-phosphate	435.251	3.095	0.013	0.005	0.019	2.449
Tetracosanoic acid	427.378	0.782	0.021	0.011	0.012	1.868
Stearoylcarnitine	426.358	2.616	0.098	0.049	0.001	2.001
Palmitic acid	255.234	0.750	89.598	63.163	0.023	1.419
cis-9-Palmitoleic acid	253.218	0.744	43.476	15.598	0.031	2.787
Azelaic acid	187.097	5.581	0.023	0.042	0.007	0.546
L-Malic acid	133.014	7.117	0.515	0.310	0.021	1.664
Hydroxyisocaproic acid	131.071	2.470	0.494	0.253	0.033	1.951
2-Hydroxy-3-methylbutyric acid	117.056	2.704	0.912	0.316	0.006	2.888

**Table 4:** Identification of different lipids detected in the scent glands during the breeding and non-breeding seasons by MALDI-MS/MS and LC-MS/MS in the positive ion mode.

Measured Value (*m/z*)	Theoretical Value (*m/z*)	Error/ppm ^1^	Molecular Formula	Precursor Type ^2^	RT (Min)	Compound Name
756.525	756.5513	3.5	C_40_H_80_NO_8_P	[M + Na]^+^	2.354	PC (32:0)
496.313	496.3325	3.9	C_24_H_50_NO_7_P	[M + H]^+^	3.026	LysoPC (16:0)

^1^ ppm represents parts per million, ^2^ M represents molecules identified by MALDI-MS.

## Data Availability

Data are available from the corresponding author upon reasonable request.

## Supplementary Materials

The following supporting information can be downloaded at: https://www.mdpi.com/article/10.3390/cells11142228/s1, Figure S1: Summary report showing the total ion chromatogram (TIC) and list of compounds detected by Gas Chromatograph Mass Spectrometer (GC-MS); Figure S2: The MS/MS spectrum of *m/z* 496.3 acquired from MALDI; Figure S3: The MS/MS spectrum of *m/z* 756.5 acquired from MALDI; Figure S4: The MS/MS spectrum of *m/z* 496.3 acquired from LC; Figure S5: The MS/MS spectrum of *m/z* 756.5 acquired from LC; Table S1: The main compounds of scent glands by GC-MS; Table S2: Differential expressed metabolites in scent glands of muskrats in the positive ion mode; Table S3: Differential expressed metabolites in scent glands of muskrats in the negative ion mode.
