# Supplementary material for: Mass Spectrometry Imaging of Lipids in the Scent Glands of Muskrat (Ondatra zibethicus) in Different Reproductive Statuses
Source: Cells. 2022 Jul 18;11(14):2228. doi: 10.3390/cells11142228 (PMC9322022; doi:10.3390/cells11142228)
Supplement: Supplementary file 1 [file cells-11-02228-s001.zip › cells-1719485-supplementary.pdf]

Supplementary data

Mass Spectrometry Imaging of Lipids in the Scent Glands of Muskrat (*Ondatra zibethicus*) in Different Reproductive Statuses

Wenqian Xie [1], Shengheng Mu [1], Jinkun Zhong [1], Chaoran Zhang [1], Haolin Zhang [1,*,†], Xiaodong Wang [2,3] and Qiang Weng [1,*,†]

1 Laboratory of Animal Physiology, College of Biological Sciences and Technology, Beijing Forestry University, Beijing 100083, China; xwq11@bjfu.edu.cn (W.X.); mushengheng@bjfu.edu.cn (S.M.); zhongjinkun2002@bjfu.edu.cn (J.Z.); zhangcr@bjfu.edu.cn (C.Z.)
2 Key Laboratory of Mass Spectrometry Imaging and Metabolomics (Minzu University of China), State Ethnic Affairs Commission Beijing 10081, China; xiaodong@muc.edu.cn
3 Centre for Imaging & Systems Biology, College of Life and Environmental Sciences, Minzu University of China, Beijing 1000081, China
* Correspondence: haolinzhang@bjfu.edu.cn (H.Z.); qiangweng@bjfu.edu.cn (Q.W.)
† These authors contributed equally to this work.

Description

As shown in Figure S1 and Table S1, the total ion chromatogram (TIC) and list of compounds were detected by Gas Chromatograph Mass Spectrometer (GC-MS).

Differential expressed metabolites in scent glands during the breeding and non-breeding seasons in the positive ion mode and negative ion mode were shown in Table S2 and Table S3.

The MS/MS spectrum of the fragment ions from MALDI and LC at *m/z* 496.3 were shown in Figure S2 and S4. Signals of ion fragments revealed the molecular structure of LysoPC (16:0).

The MS/MS spectrum of the fragment ions from MALDI and LC at *m/z* 756.5 were shown in Figure S3 and S5. Signals of ion fragments revealed the molecular structure of PC (32:0).

Table S1 The main compounds of scent glands by GC-MS

Peak#	Height%	Name
2	29.11	Cyclotridecanone
20	13.73	Z-7-Hexadecenoic acid
1	13.66	Cyclopentadecanone
12	10.33	Cyclohexanecarboxylic acid
15	7.29	11-Methyl-13-tetradecen-1-ol acetate
16	6.07	E,E-3,13-Octadecadien
3	3.48	Triacontane
11	2.89	Glycidol stearate
5	1.95	10-12-Pentacosadiynoic acid
10	1.91	Cyclohexanecarboxylic acid
4	1.73	Cycloheptadecanol
6	1.14	OleicAcid
7	1	Ethyl Oleate
8	0.95	Cyclopentadecanone
19	0.84	9-Octadecen-1-ol
17	0.84	8,11,14-Docosatrienoic acid
13	0.83	Cyclohexanecarboxylic acid
14	0.8	Cyclohexanecarboxylic acid
18	0.76	1,6,9-Tetradecatriene
9	0.68	Bicyclo[10.8.0]eicosane

Table S2 Differential expressed metabolites in scent glands of muskrats in the positive ion mode

Compound name	m/z	rt(min)	MEAN B	MEAN NB	*P* VALUE	FOLD CHANGE
PC(20:5(5Z,8Z,11Z,14Z,17Z)/20:5(5Z,8Z,11Z,14Z,17Z))	887.559	3.108	0.114	0.186	0.000	0.610
N-Tetracosanoyl-4-sphingenyl-1-O-phosphorylcholine	853.644	2.268	0.010	0.006	0.027	1.691
1,2-dioleoyl-sn-glycero-3-phosphatidylcholine	808.580	1.930	1.811	3.121	0.006	0.580
PC(16:0/16:0)	756.525	2.354	5.929	3.311	0.031	1.791
Phosphatidylethanolamine	748.571	2.122	0.167	0.097	0.025	1.723
1-Stearoyl-2-arachidonoyl-sn-glycerol	627.529	3.108	0.085	0.134	0.000	0.634
Glutathione disulfide	613.156	8.078	0.922	0.314	0.013	2.941
Naringin	545.157	2.762	0.004	0.002	0.014	2.704
1-Oleoyl-sn-glycero-3-phosphocholine	544.335	3.056	0.335	0.194	0.033	1.729
Ile-Met	542.317	3.093	0.021	0.010	0.003	2.088
1-Eicosatrienoyl-sn-glycero-3-phosphoethanolamine	504.302	3.148	0.031	0.016	0.022	1.918
LysoPC(16:0)	496.313	3.026	0.009	0.004	0.028	2.493
Suberylglycine	480.265	3.936	0.004	0.002	0.038	1.869
Glycochenodeoxycholate	467.351	3.098	0.029	0.009	0.048	3.279
1-Palmitoyl-2-hydroxy-sn-glycero-3-phosphoethanolamine	454.290	3.236	0.847	0.406	0.031	2.085
alpha-Tocopherol (Vitamin E)	431.380	0.547	0.032	0.009	0.003	3.703
Nervonic acid	430.375	2.748	0.171	0.100	0.015	1.700
Stearoylcarnitine	428.370	2.543	12.765	7.198	0.001	1.773
Theanine	387.172	4.161	0.041	0.018	0.050	2.292
Riboflavin	377.142	3.473	0.022	0.008	0.017	2.753
Suberic acid	371.176	4.182	0.003	0.001	0.045	3.158
Phe-Tyr	370.172	4.183	0.014	0.004	0.040	3.214

Lathosterol	369.347	0.557	0.111	0.041	0.008	2.729
N-Oleoylethanolamine	326.302	0.603	0.259	0.063	0.002	4.089
Linoleoyl ethanolamide	324.286	0.603	0.122	0.058	0.002	2.098
16-Hydroxypalmitic acid	314.266	0.743	0.095	0.055	0.007	1.727
Tyr-Glu	311.121	4.856	0.004	0.002	0.001	2.126
Deoxyguanosine	309.135	5.008	0.014	0.004	0.022	3.254
Arg-Met	306.152	6.688	0.006	0.003	0.043	2.319
Pro-Phe	304.158	7.405	0.009	0.005	0.039	1.761
N-Acetyl-D-Glucosamine 6-Phosphate	302.060	7.427	0.023	0.012	0.006	1.887
Palmitoyl ethanolamide	300.287	0.616	0.430	0.122	0.016	3.538
Tyr-Thr	300.162	4.862	0.005	0.002	0.023	2.758
Linoleic acid	298.271	0.603	0.552	0.303	0.046	1.821
isocarboxazid	295.115	0.616	0.030	0.019	0.002	1.559
Methoprene (S)	293.245	0.556	0.094	0.019	0.000	4.922
Argininosuccinic acid	291.127	7.660	0.058	0.028	0.032	2.092
His-Glu	285.117	6.786	0.007	0.003	0.009	2.256
N,N-Dimethylaniline	281.135	0.616	0.056	0.034	0.001	1.647
.gamma.-L-Glu-.epsilon.-L-Lys	276.152	7.719	0.028	0.017	0.005	1.660
L-Pyroglutamic acid	276.117	6.755	0.014	0.006	0.048	2.255
1,2,3-Benzenetriol	275.050	6.648	0.031	0.016	0.018	1.991
Phe-Thr	267.139	3.933	0.022	0.006	0.045	3.648
Met-Asp	265.082	6.382	0.004	0.002	0.028	2.294
L-Norleucine	263.194	4.270	0.038	0.015	0.002	2.563
2-Methylbutyroylcarnitine	246.169	3.905	23.905	3.875	0.020	6.169
Pro-Met	246.106	5.927	0.013	0.017	0.018	0.735
Gly-Lys	245.155	4.067	0.132	0.006	0.047	20.768

Pro-Glu	245.110	6.792	0.016	0.008	0.017	2.010
Monoethylglycylxylidide (MEGX)	245.105	5.406	0.752	0.502	0.043	1.498
Mimosine	243.040	7.262	0.007	0.003	0.029	1.993
p-CHLOROPHENYLALANINE	241.071	3.615	0.007	0.004	0.014	1.982
Cantharidin	238.105	2.803	0.008	0.003	0.045	2.870
Betaine	235.163	4.869	0.010	0.006	0.039	1.808
Allocystathionine	223.073	7.384	0.408	0.189	0.043	2.157
4-Hydroxy-3-methoxycinnamaldehyde	223.032	4.369	0.002	0.000	0.030	9.188
Pantothenate	220.115	4.403	0.230	0.040	0.026	5.734
2,6-Dihydroxybenzoic acid	218.040	5.135	0.004	0.008	0.042	0.522
sn-Glycerol 3-phosphoethanolamine	216.061	6.404	0.238	0.080	0.032	2.967
Leu-Ala	203.137	0.746	0.041	0.018	0.028	2.256
3-Methoxy-4-hydroxyphenylethyleneglycol	202.105	4.403	0.018	0.003	0.020	5.649
Val-Ala	189.122	0.748	0.023	0.010	0.026	2.406
DL-Indole-3-lactic acid	188.072	5.008	0.017	0.005	0.026	3.400
Phosphorylcholine	184.072	8.093	3.135	1.732	0.008	1.810
D-Mannitol	183.082	4.883	0.034	0.019	0.009	1.822
L-Tyrosine	182.079	4.886	0.248	0.141	0.021	1.760
L-Glutamine	169.056	6.062	0.094	0.079	0.030	1.191
D-Xylose	168.089	4.170	0.007	0.003	0.012	2.208
Pyridoxal (Vitamin B6)	168.063	1.624	0.010	0.004	0.012	2.417
L-Phenylalanine	166.084	4.162	0.897	0.421	0.012	2.134
trans-2-Hydroxycinnamic acid	165.053	4.886	0.127	0.068	0.023	1.882
L-Carnitine	162.111	5.746	15.577	8.454	0.004	1.843
Ribitol	153.073	3.799	0.005	0.002	0.030	2.643
L-Methionine	150.056	4.601	0.185	0.083	0.007	2.243

Diacetyl	150.054	1.836	0.043	0.015	0.002	2.925
trans-cinnamate	149.061	6.468	0.026	0.015	0.014	1.760
4-Pyridoxic acid	148.040	5.429	0.025	0.016	0.006	1.576
4-Hydroxycinnamic acid	147.042	4.884	0.021	0.011	0.032	1.959
Hypoxanthine	137.044	2.768	4.860	2.733	0.020	1.778
Perillyl alcohol	135.115	0.559	0.125	0.065	0.017	1.938
D-Aspartic acid	134.043	6.577	0.065	0.036	0.020	1.809
Ornithine	133.095	8.204	0.027	0.017	0.038	1.561
L-Asparagine	133.058	6.129	0.029	0.016	0.045	1.791
L-Leucine	132.101	4.269	0.610	0.290	0.002	2.105
N-Acetyl-L-alanine	132.063	4.115	0.012	0.005	0.038	2.302
Phenyllactic acid	131.047	4.164	0.024	0.011	0.012	2.201
Nicotinamide	123.054	0.899	5.982	2.168	0.018	2.759
Tyramine	120.079	4.164	1.472	0.674	0.013	2.185
Phenylacetic acid	119.047	4.888	0.022	0.012	0.023	1.814
Methylmalonic acid	119.033	2.768	0.082	0.046	0.012	1.762
L-Valine	118.085	4.863	0.331	0.244	0.045	1.355
Dopamine	118.063	3.914	0.060	0.054	0.005	1.125
L-Proline	116.069	5.051	2.275	1.377	0.009	1.652
N-Methylhydantoin	115.048	5.652	0.015	0.011	0.023	1.296
L-Serine	106.048	6.118	0.046	0.025	0.030	1.860
Choline	104.105	6.278	0.501	0.236	0.050	2.121
DL-2,4-Diaminobutyric acid	101.069	6.067	0.027	0.021	0.046	1.238
Pyrrolidine	72.080	4.867	0.180	0.096	0.007	1.870

Table S3 Differential expressed metabolites in scent glands of muskrats in the negative ion mode

Compound name	m/z	rt(min)	MEAN B	MEAN NB	*P* VALUE	FOLD CHANGE
2-Oleoyl-1-stearoyl-sn-glycero-3-phosphoserine	788.546	3.164	0.376	0.683	0.010	0.550
1-Palmitoyl-2-oleoyl-phosphatidylglycerol	747.517	2.309	0.026	0.061	0.000	0.427
Glutathione disulfide	611.147	8.076	1.556	0.620	0.016	2.509
L-Palmitoylcarnitine	458.348	2.652	0.610	0.225	0.002	2.718
1-Palmitoyl-2-hydroxy-sn-glycero-3-phosphoethanolamine	452.279	3.233	1.195	0.704	0.032	1.698
Fludrocortisone acetate	443.194	3.905	0.006	0.001	0.020	5.008
1-Oleoyl-sn-glycerol 3-phosphate	435.251	3.095	0.013	0.005	0.019	2.449
Glycolithocholic acid	432.314	0.660	0.175	0.052	0.038	3.352
Tetracosanoic acid	427.378	0.782	0.021	0.011	0.012	1.868
Stearoylcarnitine	426.358	2.616	0.098	0.049	0.001	2.001
Riboflavin	375.130	3.468	0.008	0.004	0.020	2.165
20-hydroxy-PGF2a	369.220	0.796	0.204	0.012	0.046	17.162
Androsterone sulfate	369.174	0.605	0.099	0.069	0.013	1.439
Pyrethrosin	343.090	6.971	0.007	0.004	0.014	1.828
Salidroside	337.062	6.488	0.011	0.015	0.015	0.714
5(S)-HpETE	335.222	0.788	0.035	0.053	0.035	0.648
Prostaglandin E1	335.222	2.661	0.039	0.008	0.026	5.001
(+-)12-HETE	319.228	0.772	0.253	0.601	0.049	0.421
N-Acetylglucosamine 1-phosphate	300.049	7.327	0.237	0.176	0.033	1.351
L-Arabinono-1,4-lactone	295.227	3.118	0.025	0.012	0.039	2.062
Palmitic acid	255.234	0.750	89.598	63.163	0.023	1.419
cis-9-Palmitoleic acid	253.218	0.744	43.476	15.598	0.031	2.787
alpha-hydroxy myristic acid	243.197	1.210	0.104	0.061	0.028	1.715

3,4,5-Trimethoxycinnamic acid	219.062	6.868	0.006	0.003	0.039	1.901
Pantothenate	218.104	4.406	0.735	0.114	0.015	6.436
L-Tryptophan	203.083	4.193	0.374	0.142	0.047	2.632
Acetylcarnitine	202.108	2.650	0.402	0.142	0.009	2.827
Propionylglycine	190.072	4.080	0.008	0.003	0.005	3.020
Azelaic acid	187.097	5.581	0.023	0.042	0.007	0.546
D-Sorbitol	181.071	4.855	0.197	0.096	0.021	2.056
L-Tyrosine	180.068	4.887	0.735	0.438	0.035	1.679
Pyridoxal (Vitamin B6)	166.051	1.614	0.065	0.036	0.011	1.805
L-Phenylalanine	164.072	4.162	1.756	0.887	0.017	1.981
L-Galactono-1,4-lactone	159.122	3.906	0.007	0.003	0.042	2.703
L-Methionine	148.044	4.595	0.109	0.049	0.015	2.240
D-Arabinono-1,4-lactone	147.030	1.343	0.081	0.025	0.018	3.263
L-Malic acid	133.014	7.117	0.515	0.310	0.021	1.664
D-Aspartic acid	132.030	6.583	0.344	0.235	0.038	1.461
Hydroxyisocaproic acid	131.071	2.470	0.494	0.253	0.033	1.951
L-Leucine	130.088	4.270	2.598	1.405	0.006	1.850
N-Acetyl-L-alanine	130.051	4.119	0.036	0.017	0.042	2.069
L-Threonine	118.051	5.728	0.082	0.048	0.012	1.692
2-Hydroxy-3-methylbutyric acid	117.056	2.704	0.912	0.316	0.006	2.888
Methylmalonic acid	117.019	1.356	0.098	0.018	0.016	5.546
L-Valine	116.072	4.866	0.501	0.298	0.013	1.682
L-Proline	114.057	5.043	0.393	0.219	0.010	1.794
L-Serine	104.036	6.117	0.144	0.095	0.049	1.520
2-hydroxy-butanoic acid	103.040	4.447	0.023	0.009	0.030	2.525
L-Alanine	88.040	6.583	0.039	0.025	0.041	1.569

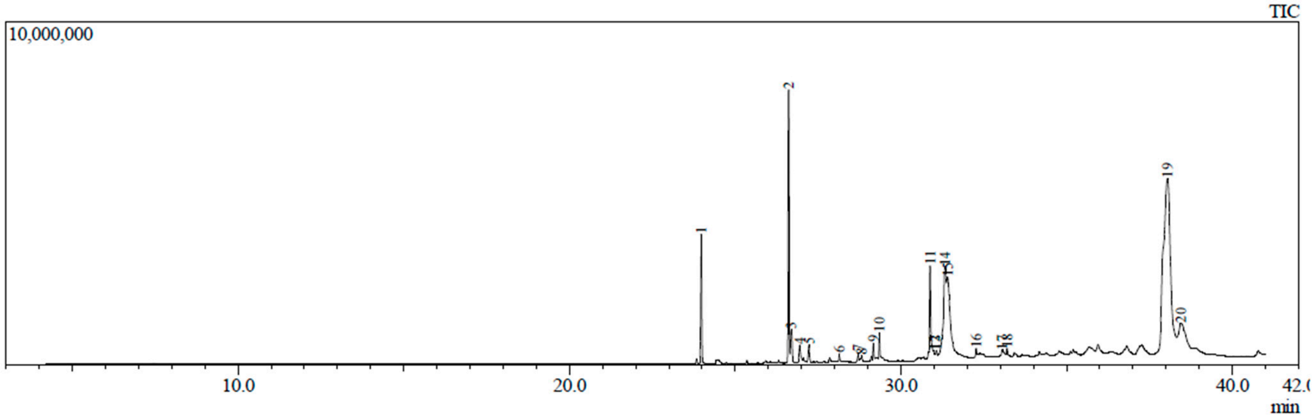

Peak Report TIC

Peak#	R.Time	I.Time	F.Time	Area	Area%	Height	Height%	A/H	Mark	Name
1	23.978	23.917	24.033	8636844	5.31	3779079	12.59	2.29		Cyclopentadecanone
2	26.613	26.558	26.658	17461468	10.73	7970540	26.55	2.19		Cyclotridecanone
3	26.690	26.658	26.767	3145376	1.93	957788	3.19	3.28	V	Tricyclo[20.8.0.0(7,16)]triacontane, 1(
4	26.948	26.892	27.025	1502948	0.92	485811	1.62	3.09		Cycloheptadecanol
5	27.225	27.167	27.292	1342243	0.82	524439	1.75	2.56		10-12-Pentacosadiynoic acid
6	28.145	28.108	28.192	456126	0.28	235323	0.78	1.94		Ethyl Oleate
7	28.706	28.667	28.742	556099	0.34	267192	0.89	2.08		1-Hydroxycyclododecanecarbonitrile
8	28.815	28.742	28.875	617832	0.38	187130	0.62	3.30	V	Bicyclo[10.8.0]eicosane, (E)-
9	29.170	29.133	29.217	973240	0.60	490722	1.63	1.98	V	Cyclohexanecarboxylic acid, undec-10
10	29.350	29.217	29.400	1404886	0.86	736041	2.45	1.91	V	Glycidol stearate
11	30.881	30.792	30.975	7323240	4.50	2710124	9.03	2.70		Cyclohexanecarboxylic acid, undec-10
12	31.000	30.975	31.033	571782	0.35	218769	0.73	2.61	V	Cyclohexanecarboxylic acid, decyl est
13	31.074	31.033	31.125	671325	0.41	209443	0.70	3.21	V	Cyclohexanecarboxylic acid, undec-10
14	31.331	31.125	31.375	16081630	9.88	2616908	8.72	6.15	V	11-Methyl-13-tetradecen-1-ol acetate
15	31.412	31.375	31.708	16082224	9.88	2289940	7.63	7.02	V	9-Cycloheptadecen-1-ol
16	32.273	32.233	32.308	457693	0.28	219143	0.73	2.09		8,11,14-Docosatrienoic acid, methyl e
17	33.050	33.017	33.175	837609	0.51	166514	0.55	5.03		7-Octylidenebicyclo[4.1.0]heptane
18	33.203	33.175	33.258	478224	0.29	203232	0.68	2.35	V	Oleyl alcohol, methyl ether
19	38.046	37.783	38.325	73734330	45.30	5003054	16.67	14.74		Z-7-Hexadecenoic acid
20	38.444	38.325	38.758	10421441	6.40	745290	2.48	13.98	V	d-Norandrostane (5.alpha.,14.alpha.)
				162756560	100.00	30016482	100.00			

Library

Figure S1. Summary report showing the total ion chromatogram (TIC) and list of compounds detected by Gas Chromatograph Mass Spectrometer (GC-MS)

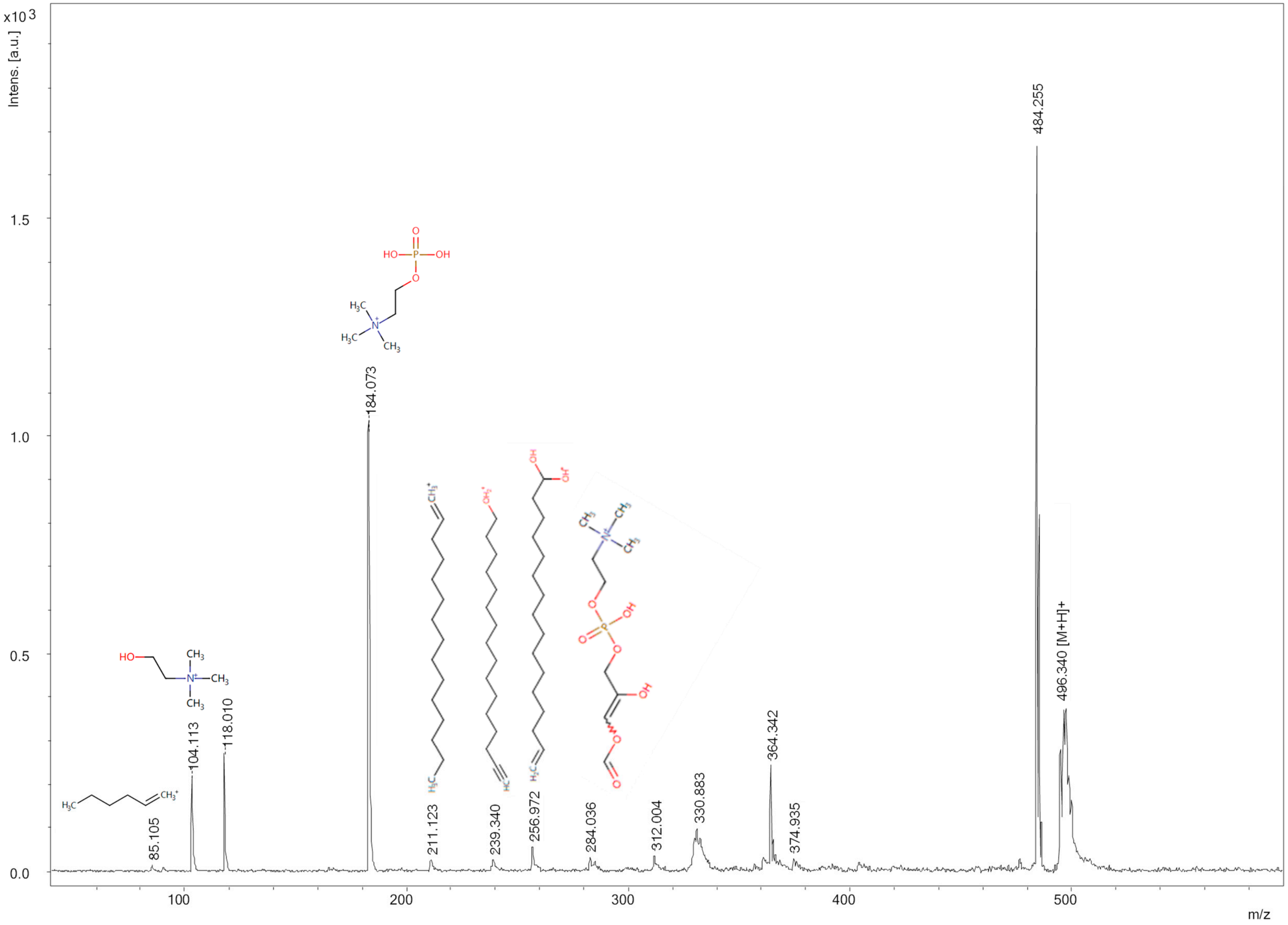

Figure S2. The MS/MS spectrum of *m*/*z* 496.3 acquired from MALDI.

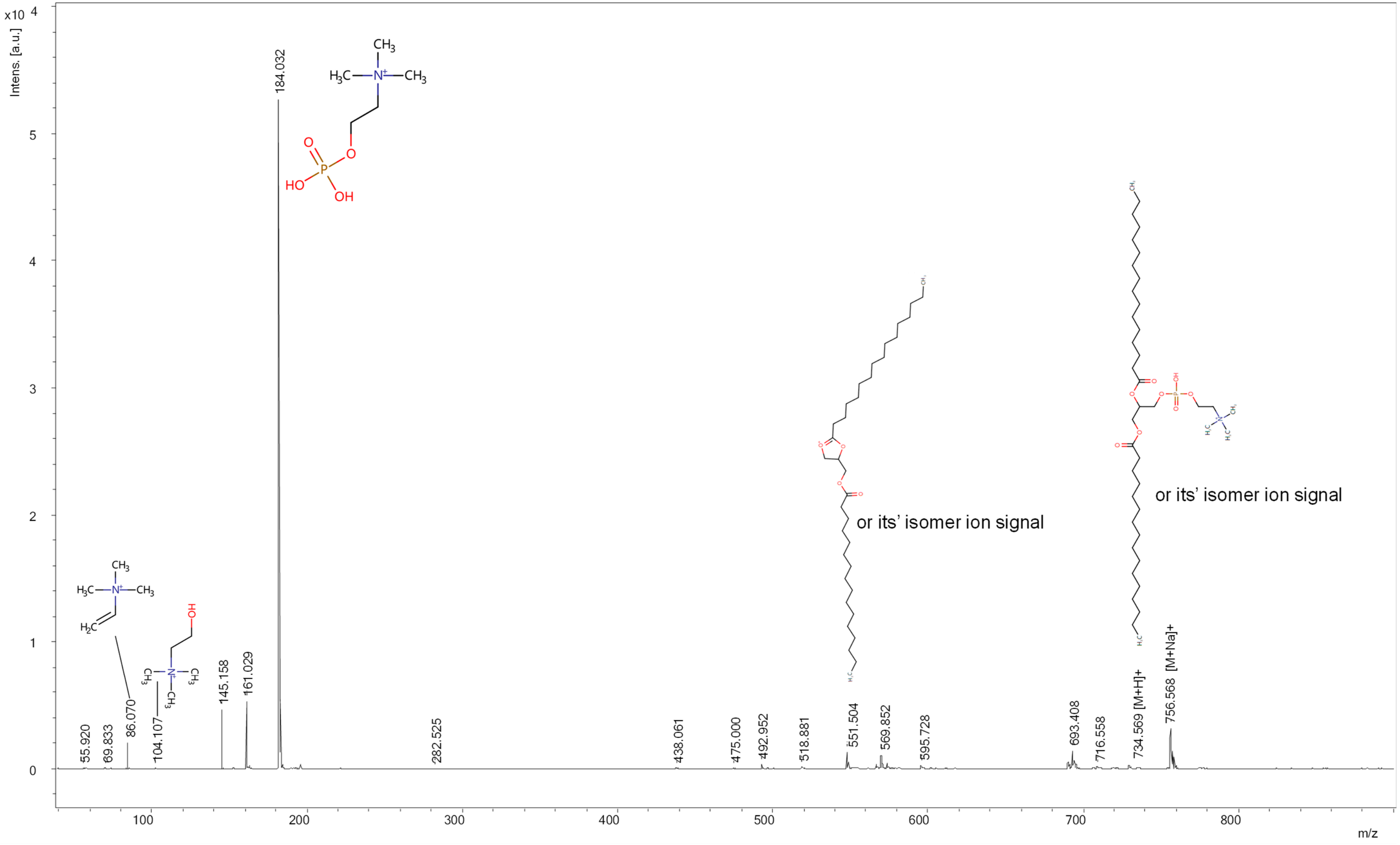

Figure S3. The MS/MS spectrum of *m*/*z* 756.5 acquired from MALDI.

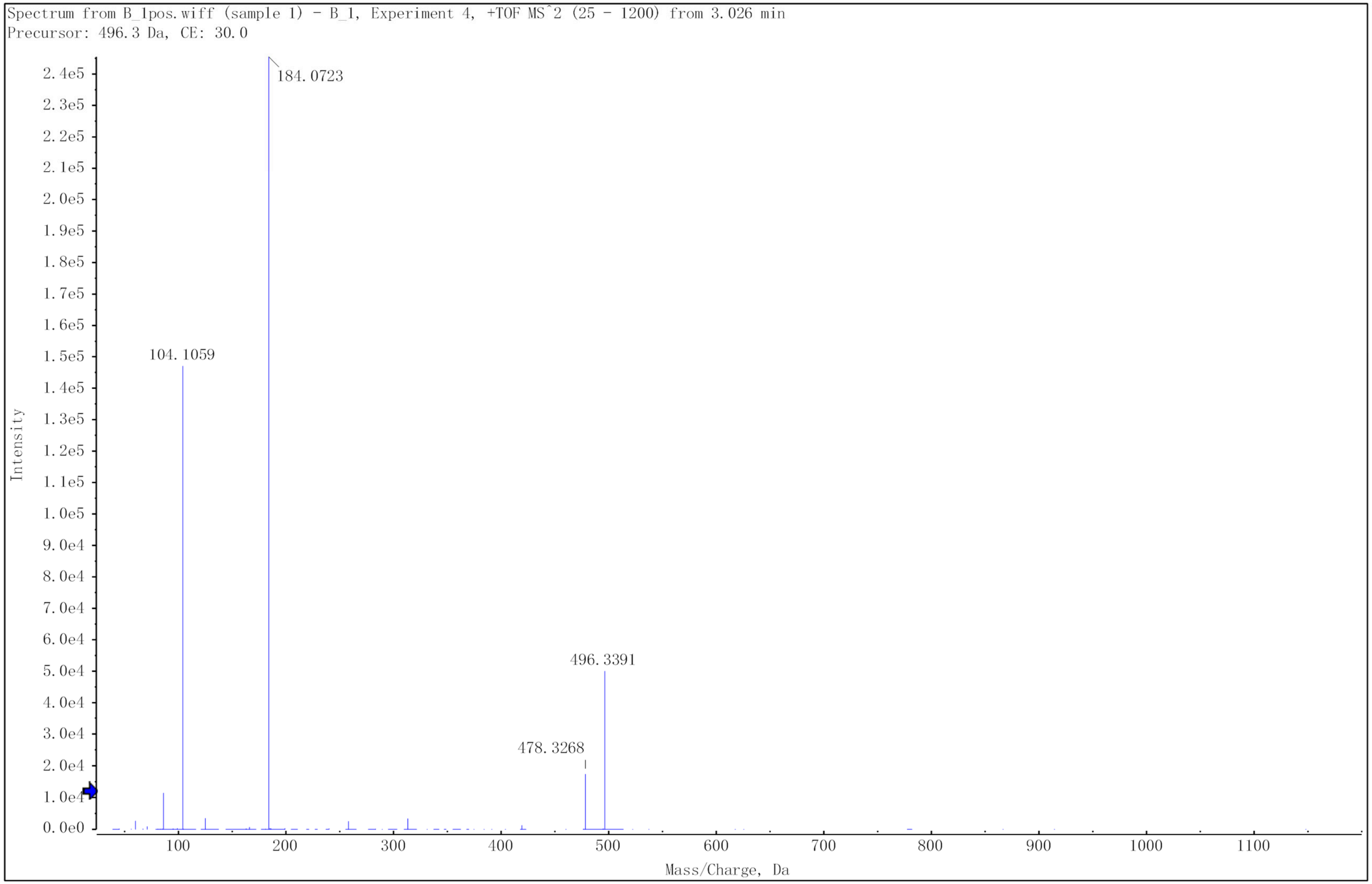

Figure S4. The MS/MS spectrum of *m*/*z* 496.3 acquired from LC.

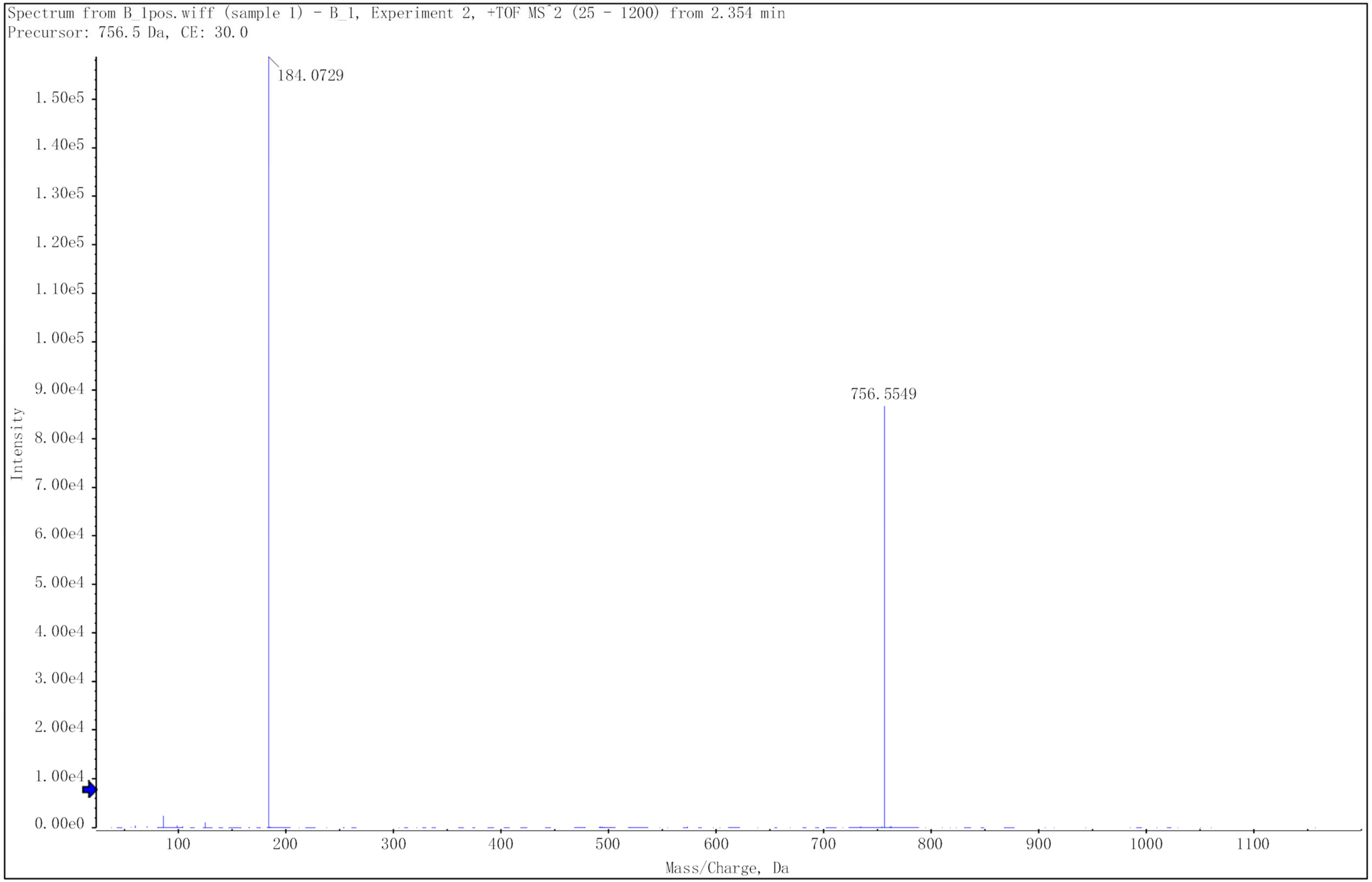

Figure S5. The MS/MS spectrum of *m*/*z* 756.5 acquired from LC.
